# The pharmacological evidence of the chang-yan-ning formula in the treatment of colitis

**DOI:** 10.3389/fphar.2022.1029088

**Published:** 2022-10-05

**Authors:** Wei Yu, Yijia Zhang, Cai Kang, Yang Zheng, Xincheng Liu, Zhenghao Liang, Jing Yan

**Affiliations:** Department of Physiology, Jining Medical University, Jining, China

**Keywords:** ulcerative colitis, macrophages, microbiota, inflammation, chang-yan-ning

## Abstract

Ulcerative colitis (UC) is a subtype of inflammatory bowel disease (IBD) and occurs mainly in the colon. The etiology of UC is rather complex and involves various pathological factors, including genetic susceptibility, dietary intakes, environment, and microbiota. In China, the Chang-Yan-Ning (CYN) formula has been utilized in the clinic to treat gastrointestinal disorders, but its pharmacological evidence remains elusive. The investigation was designed to explore the molecular and cellular mechanisms of CYN. Liquid Chromatography with tandem mass spectrometry (LC/MS) was performed to identify the key components in the formula; Network pharmacology analysis was executed to predict the potential targets of CYN; An experimental murine colitis model was established by utilizing 2% dextran sodium sulfate (DSS), and CYN was administered for 14 days. The pharmacological mechanism of the CYN formula was corroborated by *in-vivo* and *in-vitro* experiments, and high throughput techniques including metabolomics and 16S rRNA sequencing. *Results:* LC/MS identified the active components in the formula, and network pharmacology analysis predicted 37 hub genes that were involved in tumor necrosis factor (TNF), interleukin (IL)-17, hypoxia-inducible factor (HIF) signaling pathways. As evidenced by *in-vivo* experiments, DSS administration shortened the length of the colon and led to weight loss, with a compromised structure of epithelium, and the CYN formula reversed these pathological symptoms. Moreover, CYN suppressed the levels of pro-inflammatory cytokines, including IL-4, IL-1b, and TNFαin the serum, inhibited the protein abundance of IL17 and HIF-1αand increased PPARγ and CCL2 in the colon, and facilitated the alternative activation of peritoneal macrophages. While peritoneal macrophages of colitis mice enhanced reactive oxygen species (ROS) production in murine intestinal organoids, the ROS level remained stable co-cultured with the macrophages of CYN-treated mice. Furthermore, the decreased microbiota richness and diversity and the prevalence of pathogenic taxa in colitis mice were rescued after the CYN treatment. The altered metabolic profile during colitis was also restored after the therapy. We posit that the CYN therapy attenuates the development and progression of colitis by maintaining the homeostasis of immune responses and microbiota.

## Introduction

Ulcerative colitis (UC) is widely recognized as one major cause of colitis-associated cancer, which ranks third in incidence and second in term of mortality. In addition to the increased risk of developing cancer, repetitive recurrence and unpredictable clinical course of UC make a challenge for UC treatment. Despite many choices of anti-colitis drugs, it is difficult to assure good outcomes due to the complicated pathogenesis of UC, including microbiota community, diet, environment, and genetic susceptibility. In the clinic, if a widely utilized drug, such as mesalamine, fails to induce remission for patients with UC, a combined administration with steroids might be effective. However, it is known that utilizing steroids has numerous side effects. In this case, traditional Chinese medicine, which has evolved with modern medicine and already been used in the clinic for thousands of years, provides a potential alternative to treat UC as a sole drug or a complementary therapy due to its multi-component and low toxic virtue.

The Chang-Yan-Ning (CYN) formula is derived from the traditional usage of *Euphorbia humifusa* Wild. (Chinese name: Di-Jin-Cao)(DJ) and *Oldenlandia chrysotricha* (Palib.) Chun (Chinese name: Jin-Mao-Er-Cao)(EC) against gastrointestinal disorders, which is documented in a medicine encyclopedia “Zhong-Hua-Ben-Cao” and the Chinese Pharmacopoeia 2020. It is comprised of five ingredients: DJ, EC, *Cinnamomum camphora* (L.) J. Presl (Chinese name: Zhang-Shu-Gen)(ZS), *Liquidambar formosana* Hance (Chinese name: Feng-Xiang-Shu-Ye)(FX), and *Elsholtzia ciliata* (Thunb.) Hyl. (Chinese name: Xiang-Ru)(XR) (Table 1). Among the ingredients, XR is anti-inflammatory, anti-oxidative, and anti-microbial (Pudziuvelyte et al., 2020; Zhang et al., 2021), but the pharmacological investigations about other ingredients are few.

**TABLE 1:** Components in the *Chang-Yan-Ning* (CYN) formula.

Scientific name of the herb	Chinese name	Material	Weight (%)
*Euphorbia humifusa* Wild.	Di-Jin-Cao	Leaf	23
*Oldenlandia chrysotricha* (Palib.) Chun	Jin-Mao-Er-Cao	Leaf	31
*Cinnamomum camphora* (L.) J.Presl	Zhang-Shu-Gen	rootstalk	23
*Liquidambar formosana* Hance	Feng-Xiang-Shu-Ye	Leaf	12
*Elsholtzia ciliata* (Thunb.) Hyl.	Xiang-Ru	rootstalk	12

Taking advantage of emerging network pharmacology and high throughput technologies, we identified the components of the CYN formula and predicted their targets involved in UC, and validated the anti-colitis efficacy by examining inflammation, hyper-oxidation, and microbiome profile.

## Materials and methods

### Chang-yan-ning preparation and validation

Precessed 660 g DJ, 900 g EC, 660 g ZS, 330 g XR, and 330g FX powder were extracted with water at 95°C for 2 h, and approximately 280 g extraction was obtained. 0.2 g extraction was subjected to LC-30 (Shimadzu)-Hybrid Quadruple time-of-flight mass spectrometer (TOF-MS) with electrospray ionization source (ESI) was utilized to identify the components of CYN as the previous protocol (Yan et al., 2021).

### Network pharmacology

Based on the validated components by TOF-MS, the targets of those components, which are above 20% oral bioavailability and 0.18 drug-likeness, were retrieved from the Lab of Systems Pharmacology database (Ru et al., 2014). A component-target network with UC-associated genes collected from GeneCards (Rebhan et al., 1997; Safran et al., 2010), DrugBank (Wishart et al., 2018), Online Mendelian Inheritance in Man (OMIM) (Hamosh et al., 2002), PharmGkb (Whirl-Carrillo et al., 2012), and Statistics of Therapeutic Target Database (TTD) (Wang et al., 2020) was built. According to the Protein-protein interaction (PPI) network with a confidence score ≥ 0.7 acquired from the Search Tool for Retrieval of Interacting Genes/Proteins database (Szklarczyk et al., 2019), the hub genes were calculated by CytoNCA package with indices of betweenness centrality (BC), closeness centrality (CC), degree centrality (DC), local average connectivity (LAC), eigenvector centrality (EC), and network centrality (NC) (Tang et al., 2015). Gene Ontology (GO) and Kyoto Encyclopedia of Genes and Genomes (KEGG) pathway analysis were performed by the R tool (Ashburner et al., 2000).

### Experimental murine colitis models and chang-yan-ning treatment

All experiments were performed under the guidelines of the Institutional Animal Care and Use Committee of Jining Medical University in China (SYXK-Shandong province-2018-0002) (THE ethical approval was from 20^th^ March 2020 to 8^th^ February 2023). Mice were anesthetized by Isoflurane (RWD life science, Shenzhen City, China) and sacrificed.

C57BL/6N mice (Pengyue animal center of Shandong province, China) (male, about 20 g, 8 weeks old) were treated with 2% Dextran sodium sulfate (DSS, MP Biomedicals) for 7 days (Okayasu et al., 1990), followed by 14-day CYN therapy at two concentrations. As suggested in the Chinese Pharmacopoeia 2020, it is the dose of 96 mg/kg each day for the human being, which is equal to 1.2 g/kg for mice. Therefore, a high (24 mg per day), or low (12 mg per day) dose of CYN extraction was given to the mice subjected to experimental colitis, and mesalamine (200 mg/kg) (MedChemExpress, China) and 0.9% saline were used as a positive drug and negative control, respectively (Kucharzik et al., 2020). Every 8 mice were grouped. The disease active index (DAI) were calculated with the following criteria: weight loss (weight gain or no loss-0; 1∼2 g: 1; 2∼4 g: 2; 4∼5 g:3; >5 g: 4); feces (healthy: 0; soft: 1; watery: 2; liquid: 3); bloody stool test (no blood within 2 min: 0; positive in 10 s: 1; light purple within 10 s: 2; heavy purple within 10 s: 3)(Leagene, China); (Yan et al., 2021).

Colon samples were fixed by paraformaldehyde (4% PFA) and embedded. The cut slice was stained with hematoxylin and eosin (H&E)(Nikon DS-U3, Pannoramic 250FLASH) and determined by histological scores: epithelial destruction (multiple sites:3, one site:1, null: 0), immune cells infiltrates (multiple sites:3, one site:1, null: 0), crypt loss (multiple sites:3, one site:1, null: 0).

### Enzyme-linked immunosorbent assay

ELISA kits (Abcam technology, United States) were utilized to examine cytokine levels in serum: Mouse IL-4 (Sensitivity: 1 pg/ml, Range: 1 pg/ml - 200 pg/ml), IL-1b (Sensitivity: 1 pg/ml, Range: 1 pg/ml - 100 pg/ml), and TNFα(Sensitivity: 60 pg/ml, Range: 94 pg/ml - 6,000 pg/ml). The intensity of the color was acquired at 450 nm.

### 16S rRNA sequencing

The feces and mucus within the colon were collected and mixed and processed by 16S rRNA sequencing. Raw data were filtered (Bolger et al., 2014; Kechin et al., 2017) and produced paired-end reads. Corrected paired-end reads generated Circular Consensus Sequencing (CCS) reads. After the removal of chimeras, OTU (operational taxonomic unit) analysis was performed with a similarity > 97%. Tax4Fun and Bugbase analysis was conducted to predict the alterations in KEGG pathways. Raw data is available with the accession number in SRA (PRJNA827781).

### Untargeted metabolomics

Feces and mucus sample was treated with methanol and standard internal substances. After the ultrasound and frozen treatment, the samples were centrifugated. Supernatant was subjected to ultra-high-performance liquid chromatography (UHPLC) coupled with TOF-MS (Garcia and Barbas, 2011). Acquisition software (Analyst TF 1.7, AB Sciex) was utilized to assess the full scan survey MS data, which were then converted by ProteoWizard and generated the retention time (RT), massto-charge ratio (m/z) values, and peak intensity. According to the In-house MS2 database, substances were identified and determined by Orthogonal projections to latent structures- discriminant analysis (OPLS-DA). The cut-off is *p* value < 0.05, fold change (FC) > 1, and Variable Importance in the Projection (VIP) > 1. The higher Q2 value (more than 0.5) indicates a strong predictivity of the model. In the permutation test, the original models of R2 and Q2 that are higher than all the permutated models with a vertical axis intersection of Q2 (zero or lower than zero) represent the validity of OPLS-DA. HMDB (Human Metabolome Database) and the KEGG database were utilized for the annotation of metabolites.

### Macrophage (Mφ) culturing

Abdominal liquids from mice were collected and centrifuged, and diluted in the complete RPMI 1640 medium (10% fetal bovine serum, 1% Penicillin-Streptomycin Solution).

### Intestinal organoid culture and co-culture system

Small intestine was cut into small pieces and washed 30 times. After digestion, the liquid was filtered and centrifugated. The cells were cultured in the complete growth medium for organoids (Stemcell Technologies, Canada). The intestinal organoids (IOs) were subjected to experiments on day 9 (Yu et al., 2020). To measure the production of reactive oxygen species (ROS), peritoneal *Mφs* were put in the upper chamber of a 0.3 μm diameter transwell (Corning, United States), and co-cultured with IOs for 24 h, followed by 30 min of MitoSOX™ Red Mitochondrial Superoxide Indicator)(Thermo Fisher, United States) treatment. Cells were mounted on the fluorescent microscope (Zeiss) at 590 nm.

### Western blotting

Total protein was subjected to SDS-PAGE (sodium dodecyl sulfate-polyacrylamide gel electrophoresis) and transferred. After the blockage, antibodies (IL-17, PPAR-γ, CCL2S, HIF-1α, Arg1, IL-4, CYP1A1, CYP3A4, GAPDH) were administered. After 20–24 h, secondary antibodies conjugated with horseradish peroxidase (HRP) were added for 2 h. Protein abundance was visualized by enhanced chemiluminescence substrate. All unclaimed chemical reagents were bought from Thermo Fisher, United States.

### Statistical analysis

For two groups, an unpaired Student t-test was performed. For multiple comparisons, ANOVA was utilized. All charts were presented as means ± standard deviation. A *p*‐value < 0.05 was considered statistically significant.

## Results

### Network pharmacological prediction of the anti-colitis efficacy of the chang-yan-ning formula

**FIGURE 1:**
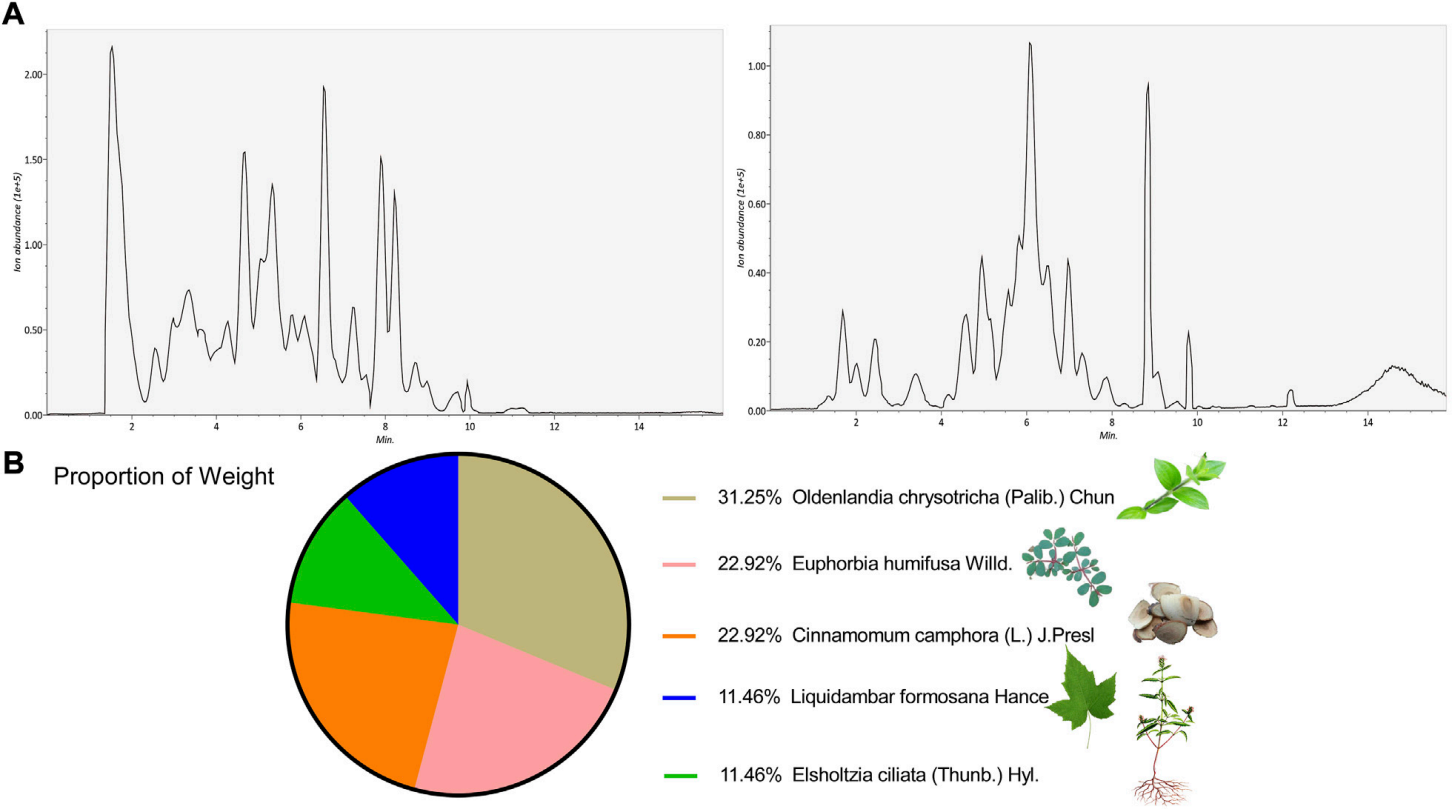
LC-MS/MS identified active components of the Chang-Yan-Ning formula. LC-MS/MS validation of the components in the CYN formula **(A)**; **(B)** The weight proportion of the formula.

**FIGURE 2:**
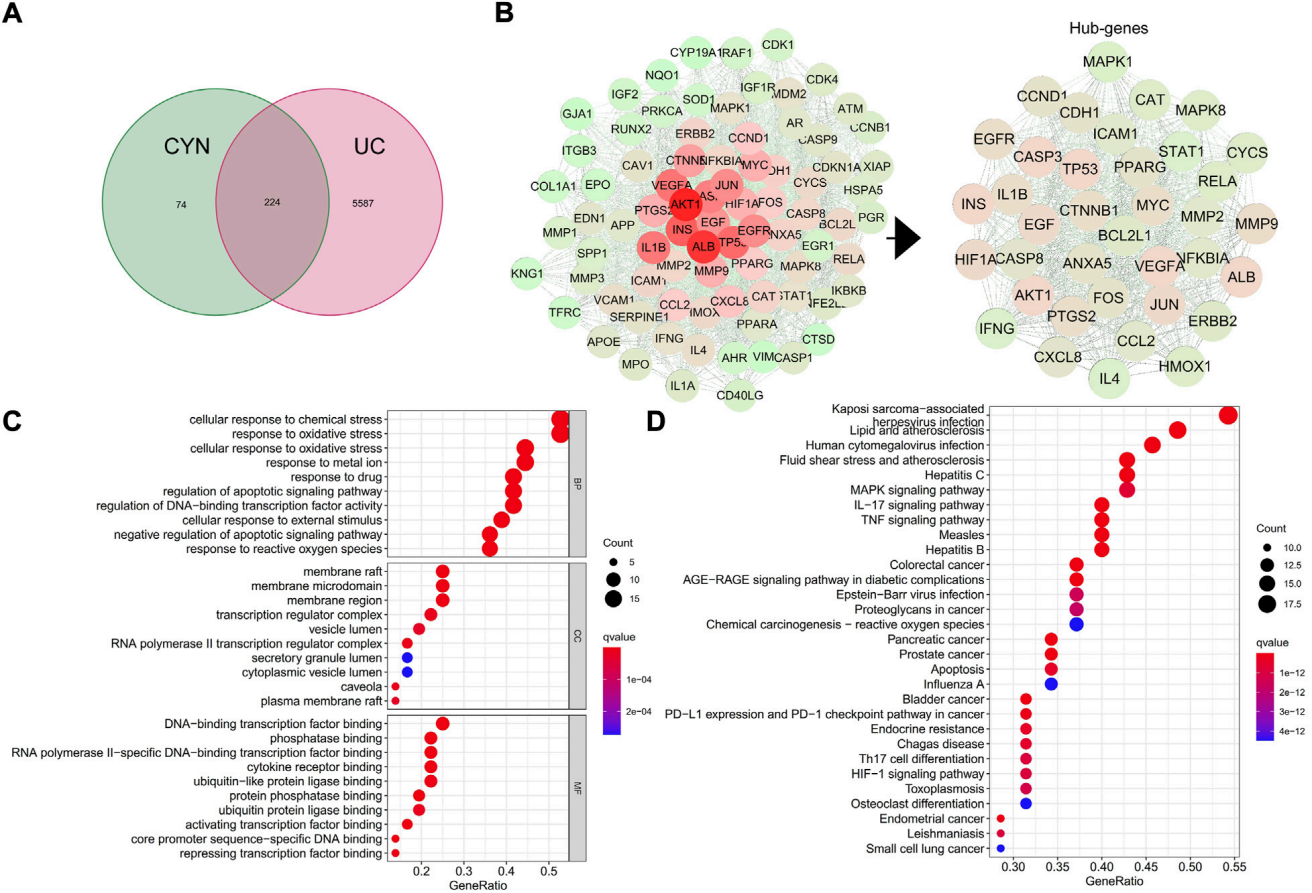
Network pharmacology analysis. The shared targets of CYN and ulcerative colitis (UC) **(A)**; Hub genes of the CYN to combat UC **(B)**; GO analysis **(C)** and KEGG analysis **(D)** of the hub genes.

To identify the key active components in the formula, LC-MS analysis was executed. The core active components, such as gallic acid and methylgallate in DJ, catechol in XR, asperulosidic acid in EC, kaempferol and quercetin, were confirmed (Figure 1A) (Supplementary Table S1). The CYN formula is composed of five ingredients, EC took up 31% and thus played a central role, while DJ and ZS were responsible for 46% of the total weight (Figure 1B). There were 224 shared genes between CYN and UC (Figure 2A) (Supplementary Table S2). Among these targets, 37 genes were considered hub genes that were above the median values of BC-16.721506515, CC-0.704347826, DC-47, LAC-36.02462121, EC-0.104429837, and NC-40.10848235 (Figure 2B), which were associated with cellular response to oxidative stress, reactive oxygen species (ROS), and external stimulus predicted by GO analysis (Figure 2C), and were enriched in IL-17, TNF, HIF-1 signaling pathways, and colorectal cancer (Figure 2D).

### Chang-yan-ning suppresses dextran sodium sulfate-induced experimental colitis progression

**FIGURE 3:**
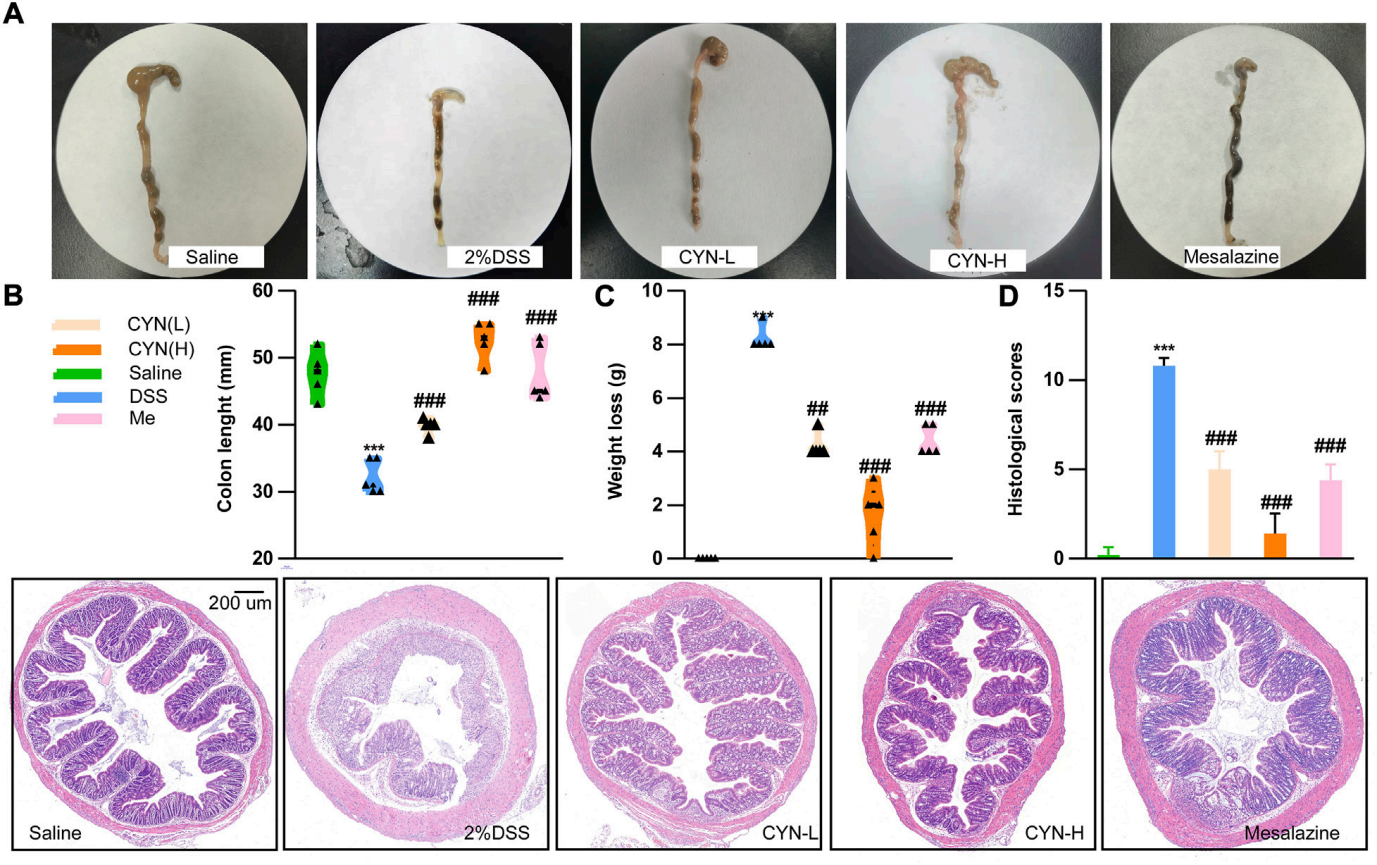
The attenuated colitis progression of experimental murine colitis mice after CYN treatment. Colon samples from experimental murine colitis models subjected to different concentrations of CYN (high:24 mg, low:12 mg per day), or mesalazine (200 mg/kg) **(A)**; The length of colon **(B)**, weight loss **(C)**, HE staining and histological scores **(D)**. *indicates a significant difference from the saline group, # indicates a significant difference from the DSS group.

To corroborate the anti-colitis effect predicted by network pharmacology, a murine colitis model was constructed by feeding 2% DSS water to mice for 7 days, followed by 14 days of CYN treatment. Both doses of CYN alleviated DSS-induced colitis. Shrinking caecum, bloody stool (Figure 3A), shortened colon length (Figure 3B), as well as weight loss (Figure 3C) were rescued after CYN treatment. Noteworthy, the high dose of CYN showed superiority over mesalamine combating bloody stool, caecum, and weight loss. H&E staining showed that DSS led to loss of crypts and goblet cells, neutrophil infiltrates, and swollen epithelium, and all these pathological symptoms were reversed by CYN. Compared with the mesalamine administration, the colon samples in the CYN group had fewer neutrophil infiltrates (Figure 3D).

### Chang-yan-ning inhibits inflammation and inflammation-associated reactive oxygen species production

**FIGURE 4:**
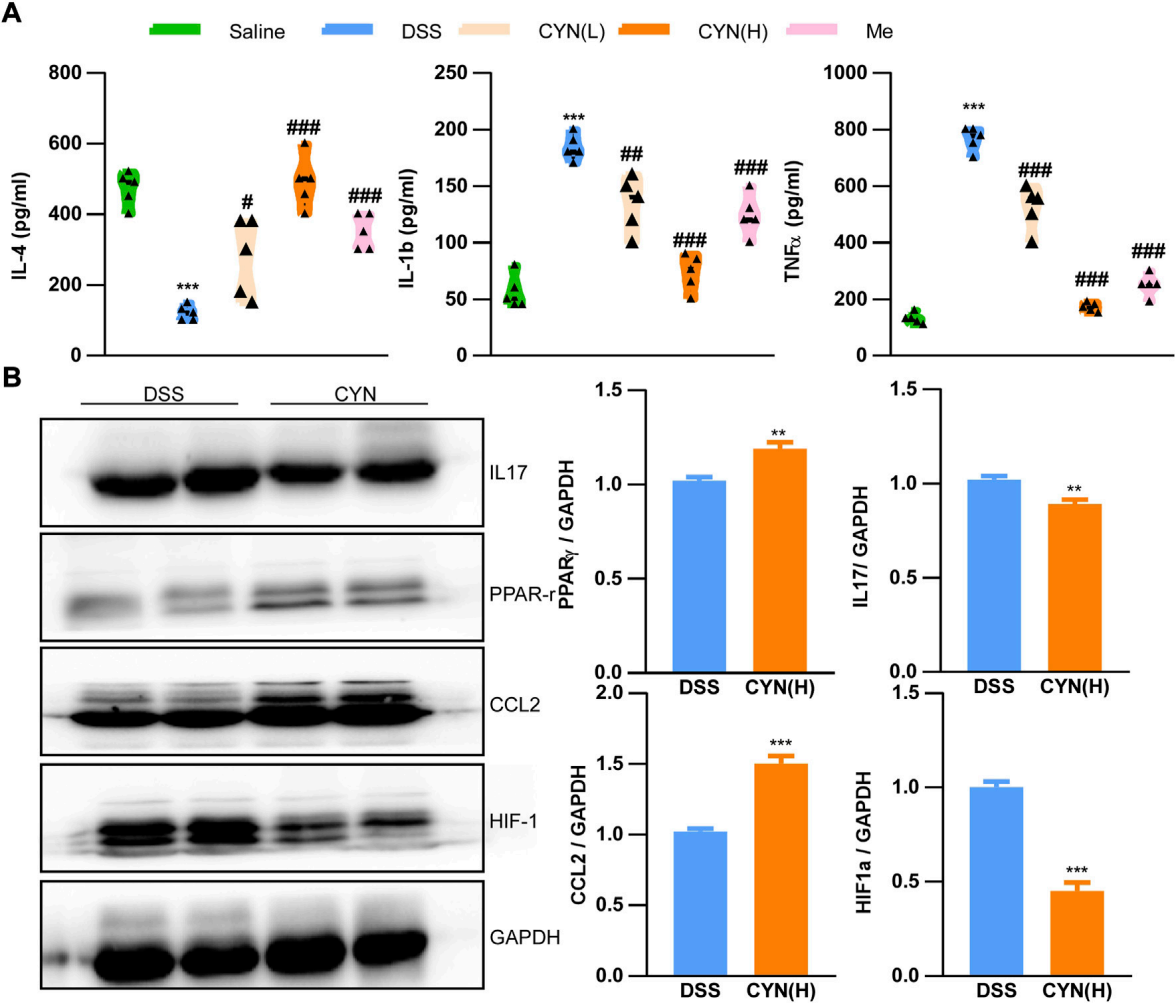
The rescued pathological cellular mechanisms by CYN adminstration. The levels of IL-4, IL-1b, TNFα in the serum of colitis mice after different doses of CYN, or mesalazine **(A)**; The protein abundance of IL17, PPARγ, CCL2, and HIF-1α in the colon of colitis mice subjected to a high dose of CYN. *, # indicates a significant difference from the saline and DSS group, respectively **(A)**; *indicates a significant difference from the DSS group **(B)**.

**FIGURE 5:**
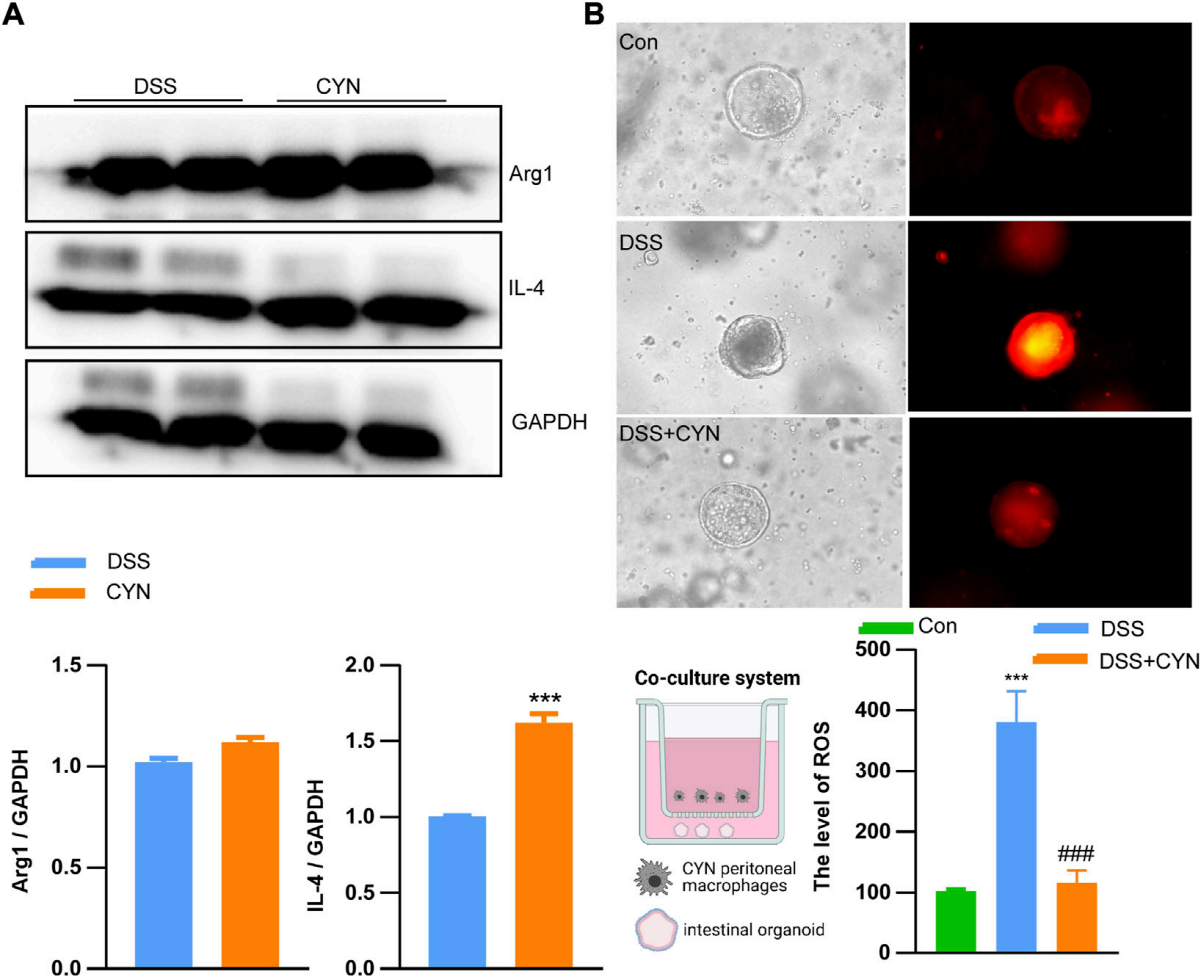
CYN favors the alternative activation of peritoneal macrophages. The protein abundance of Arg1 and IL-4 in peritoneal macrophages of colitis mice after a high dose of CYN treatment **(A)**; The reactive oxygen species level in intestinal organoids pre-treated with the peritoneal macrophages of colitis mice after the high dose of CYN **(B)**.*indicates a significant difference from the DSS group **(A)**; *, # indicates a significant difference from the control and DSS group, respectively **(B)**.

According to the predicted targets of network pharmacology, CYN influences various inflammation-associated signaling pathways, including IL-4, IL-1b, TNFα, IL-17, HIF-1α, PPAR-γand CCL2. In this context, the serum levels of IL-4, IL-1b, and TNFαwere examined, and we found that these increased cytokines in colitis mice were suppressed by CYN and mesalamine, as evidenced by ELISA experiments (Figure 4A). Moreover, the high dose of CYN reduced the expression of IL-17 and HIF-1αin the colon samples from colitis mice, whereas it facilitated the protein abundance of PPAR-γand CCL2 (Figure 4B). Furthermore, the high dose of CYN enhanced the alternative activation of peritoneal macrophages isolated from colitis mice, as shown by the increased expression of IL-4 and Arg1 (Figure 5A). Consistently, the intestinal organoids were co-cultured with peritoneal macrophages of colitis mice and exhibited a considerably higher ROS level, and this elevation was not observed when organoids were treated with the macrophages of the CYN group (Figure 5B), implying an inflammation-suppressive immune status after CYN treatment.

### Chang-yan-ning restores the homeostasis of gut microbes and their metabolites

**FIGURE 6:**
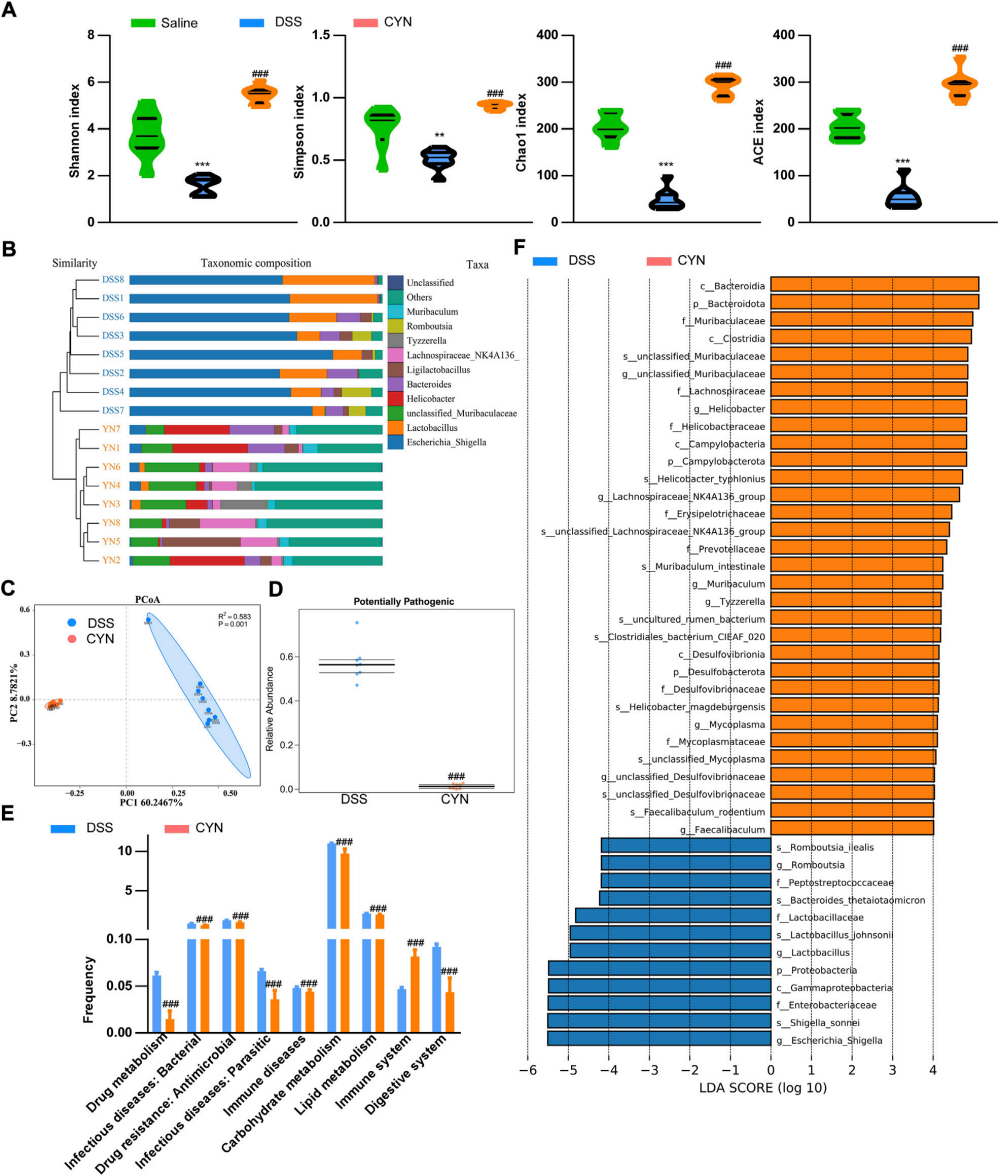
Improved microbiota profile in colitis mice after CYN treatment. The indices of alpha-diversity **(A)**, the taxa composition of the microbiota **(B)** in the fecal samples from colitis mice subjected to a high dose of CYN therapy; PCoA demonstrating cluster distributions **(C)**, BugBase analysis **(D)** and picrust2 analysis predicting the influenced functions and KEGG pathways by CYN **(E)**, and LEFSe analysis **(F)** of the differentially expressed microbiota between the DSS group and CYN treatment group. *, # indicates a significant difference from the saline and DSS groups, respectively.

Dysbiosis correlates with clinical outcome and recurrence. DSS treatment reduced the richness and diversity of the gut microbiota, as reflected by chao1 and ACE, Shannon and Simpson indices, which was reversed after the high dose of CYN (Figure 6A) (Supplementary Table S3). By Bray Curtis analysis, CYN treatment markedly influenced the taxonomic composition (Figure 6B). Principal Co-ordinates Analysis (PCoA) showed that the CYN group clustered distinctly from the DSS group (Figure 6C). The enhanced prevalence of phylum *Proteobacteria* and CAC-associated genus *Escherichia Shigella* in colitis mice was suppressed after the administration of CYN (Figure 6F) (Supplementary Figure S1). Bugbase analysis demonstrated a decline in potentially pathogenic taxa in the CYN group (Figure 6D), and Tax4Fun pointed out that the therapy suppressed infection and immune diseases, and influenced multiple pivotal metabolic pathways, including drug metabolism, lipid and carbohydrate metabolism (Figure 6E).

**FIGURE 7:**
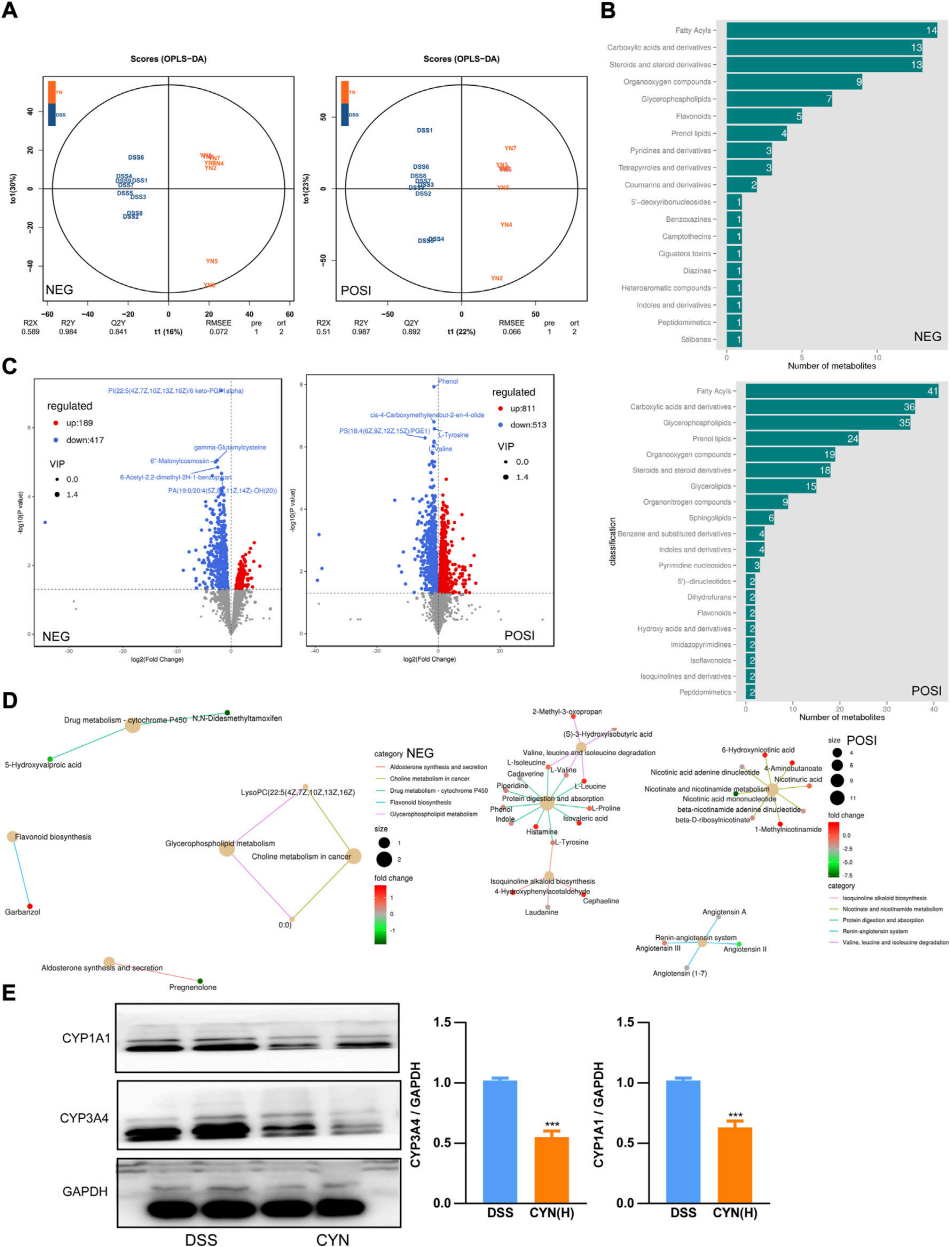
The reversed metabolic profile in colitis mice after CYN treatment. OPLS-DAOPLA-DA **(A)**, HDMB enrichment **(B)**, volcano chart **(C)** showing the metabolite clusters under positive and negative mode; KEGG pathways **(D)**. Western blotting showing the protein levels of CYP1A1 and CYP3A4 in colon samples from colitis mice after the high dose of CYN administration **(E)**. *indicates a significant difference.

Consistent with the restored microbiota profile, OPLS-DA showed that in both negative and positive modes, the metabolites of the saline, DSS, and CYN groups clustered distinctly (Figure 7A) (Supplementary Figure S2A). The differentially expressed metabolites with a VIP>1 were fatty acyls, carboxylic acid, and glycerophospholipids based on HMDB (Figures 7B,C) (Supplementary Table S4). KEGG analysis predicted that the induction of CY450 activity in DSS mice was inhibited by the CYN treatment, which was in line with the results of 16S rRNA gene sequencing (Figure 7D) (Supplementary Figure S2B). Hence, protein extraction of colon samples was immunoblotted with multiple antibodies that recognize proteins responsible for drug metabolism, and we confirmed that CYN considerably suppressed CYP3A4 and CYP1A1 (Figure 7E).

## Discussion

In this study, the mechanisms underpinning the anti-colitis effect of the CYN formula have been delineated. In term of the five ingredients in CYN, XR can maintain the homeostatic status of the immune system, microbiota, and ROS production (Pudziuvelyte et al., 2020; Zhang et al., 2021), but little is known about the therapeutic effects of DJ, EC, ZS, and FX. Assuming the limitation, LC-MS was performed to identify the active components of the formula and their potential targets were predicted by network pharmacology analysis. Among the 224 shared genes between UC and CYN, 37 hub genes were retrieved and KEGG analysis demonstrated the involved oxidation, infection, and inflammation-regulated reactions. Herewith, we delineated the pharmacological mechanisms of the CYN from the aspects of inflammation, ROS, and microbiome.

First, excessive inflammatory responses exacerbate the compromised colon epithelium, with consequent mucosal lesions and colonic erosion. By referring to the retrieved hub genes and their influenced pathways, IL-1β, IL-17 (Yin et al., 2021), TNFα (Xiao et al., 2016), and HIF-1α (Bäcker et al., 2017; Kerber et al., 2020) are pro-inflammatory in colitis mice, and IL-4 (Callejas et al., 2021), CCL2 (Maharshak et al., 2010), and PPARγ (Zhao et al., 2018) are conducive to remission. The increased IL-1b and TNF serum levels and enhanced HIF-1 and IL-10 expression in colitis mice were suppressed by CYN therapy, and the treatment facilitated IL-4, CCL2, and PPARγ expression. IL-4 and CCL2 drive the alternative polarization of macrophages (Sierra-Filardi et al., 2014; Shapouri-Moghaddam et al., 2018), which plays a pivotal role in the resolution of inflammation and favors wound healing. Consistently, CYN increased the protein abundance of IL-4 and Arg1 in peritoneal macrophages, indicating an alternative activation.

When it comes to the regulation of ROS, we observed that macrophages isolated from the DSS group aggravated the ROS production in intestinal stem cells due to the release of inflammation-promoting cytokines by macrophages (Yan et al., 2021; Yu et al., 2021). Since intestinal stem cells are responsible for epithelial replenishment, excessive ROS-induced cell death would exacerbate the compromised epithelial integrity during colitis. The macrophages of CYN-treated colitis mice did not instigate ROS production in stem cells, which at least in part suggests an orchestrated immune microenvironment in CYN mice.

Third, not only is dysbiosis a causal factor for the pathogenesis of UC, it also determines whether a treatment achieves a complete remission. DSS increased the prevalence of potentially pathogenic taxa in the murine gut, with elevated colonization of the genus *Escherichia Shigella*, which induces diarrhea and provokes acute colitis (Belotserkovsky and Sansonetti, 2018), and this induction was suppressed by the CYN treatment. Moreover, the therapy facilitated the relative abundance of *Lachnospiraceae NK4A136* and *Faecalibaculum rodentium*, which enhances gut barrier function (Ma et al., 2020; Stadlbauer et al., 2020) and protects from colorectal cancer (Hindson, 2020), respectively, pointing to an improvement in gut flora composition. According to KEGG analysis, the CYN treatment decreased DSS-induced risks of developing infection and inflammation, and established a metabolic profile characteristic for suppressed cytochrome P450 activity. Increased CYP450 expression would produce excessive reactive metabolites and instigate intestinal immune responses, thereby increasing susceptibility to UC (Plewka et al., 2014; Sen and Stark, 2019). In support of the decreased cytochrome P450 activity in metabolomic result, we observed that CYP3A4 and CYP1A1 protein abundance was markedly reduced in the colon samples of colitis mice subjected to the CYN therapy.

No other organ like the gut sustains such challenges from the external environment and microorganisms, and immune tolerance has a fundamental role in maintaining an inappropriate immune status. Under physiological conditions, the monolayer of intestinal epithelial cells, as the first defensive barrier against pathogen invasion, undergo orchestrated cellular death that has evolved to benefit the host and are continuously replenished by intestinal stem cells. In the context of UC, sustained inflammation exacerbates ROS production in intestinal stem cells and other intestinal cell types, which culminates in excessive programmed cell death in the absence of an actual need with a consequent epithelial breach, allowing the colonization of pathogenic taxa and subsequent epithelial erosion (Weingarden and Vaughn, 2017). The CYN formula counteracts intestinal pathology by efficaciously promoting the resolution of inflammation and excessive ROS abundance, thereby preventing dysbiosis. Among the ingredients, EC is the major ingredient and its iridoid-asperulosidic acid alleviates oxidative stress and inflammation (Peng et al., 1999; Wu et al., 2022); ZS is anti-inflammatory (Li et al., 2018; Xiao et al., 2021; Bampidis et al., 2022) and the gallic acid from DJ protects against colitis by its high antioxidant, anticancer, anti-microbial effects (Pandurangan et al., 2015; Gao et al., 2016; Al Zahrani et al., 2020). XR is considered a potent anti-colitis alternative with solid experimental evidence about its anti-inflammatory, antinociceptive, antioxidant, anti-microbial and anticancer activities (Zhang et al., 2021). Herewith, all the five ingredients are anti-inflammatory and anti-oxidant, while the re-establishment of microbiota homeostasis is accomplished by XR and DJ. Last but not least, FS shows a potent anti-neoplastic property (Zhong et al., 2016; Qian et al., 2020). Our experiments showed that the combination of these five ingredients exerted a potent anti-colitis effect by orchestrating the homeostasis of inflammation and microbiota. However, we are aware of the limitations in the study, and further investigations into pharmacokinetics are needed.

## Conclusion

We propose that the CYN formula is an effective anti-colitis therapy, the underlying pharmacological mechanisms of which involve anti-inflammation and anti-oxidation, as well as the re-establishment of a healthy microbiota community.

## Data Availability

The data presented in the study are deposited in the SRA repository, accession number PRJNA827781.

## Supplementary material

Supplementary Table S1 LC-MS/MS identified active components of CYN.

Supplementary Table S2 The shared genes.

Supplementary Table S3 Alpha diversity indices of the gut microbiota after CYN treatment.

Supplementary Table S4 The altered metabolites in the fecal samples from colitis mice.

Supplementary Figure S1 The differentially colonized microbiota between the saline and DSS groups.

Supplementary Figure S2 The altered metabolites in the fecal samples from colitis mice OPLS-DAOPLA-DA **(A)** showing the metabolite clusters between saline and colitis mice under positive and negative mode; KEGG pathways **(B)**.

## Abbreviations

IBD, inflammatory bowel disease; CYN, Chang-Yan-Ning; UC, ulcerative colitis; CAC, colitis-associated carcinogenesis; CC, closeness centrality; BC, betweenness centrality; DC, degree centrality; EC, eigenvector centrality; LAC, local average connectivity; NC, network centrality; GO, Gene Ontology; KEGG, Kyoto Encyclopedia of Genes and Genomes; CYP, cytochrome.
