# Supplementary figures and images for: The pharmacological evidence of the chang-yan-ning formula in the treatment of colitis
Source: Front Pharmacol. 2022 Oct 5;13:1029088. doi: 10.3389/fphar.2022.1029088 (PMC9579319; doi:10.3389/fphar.2022.1029088)

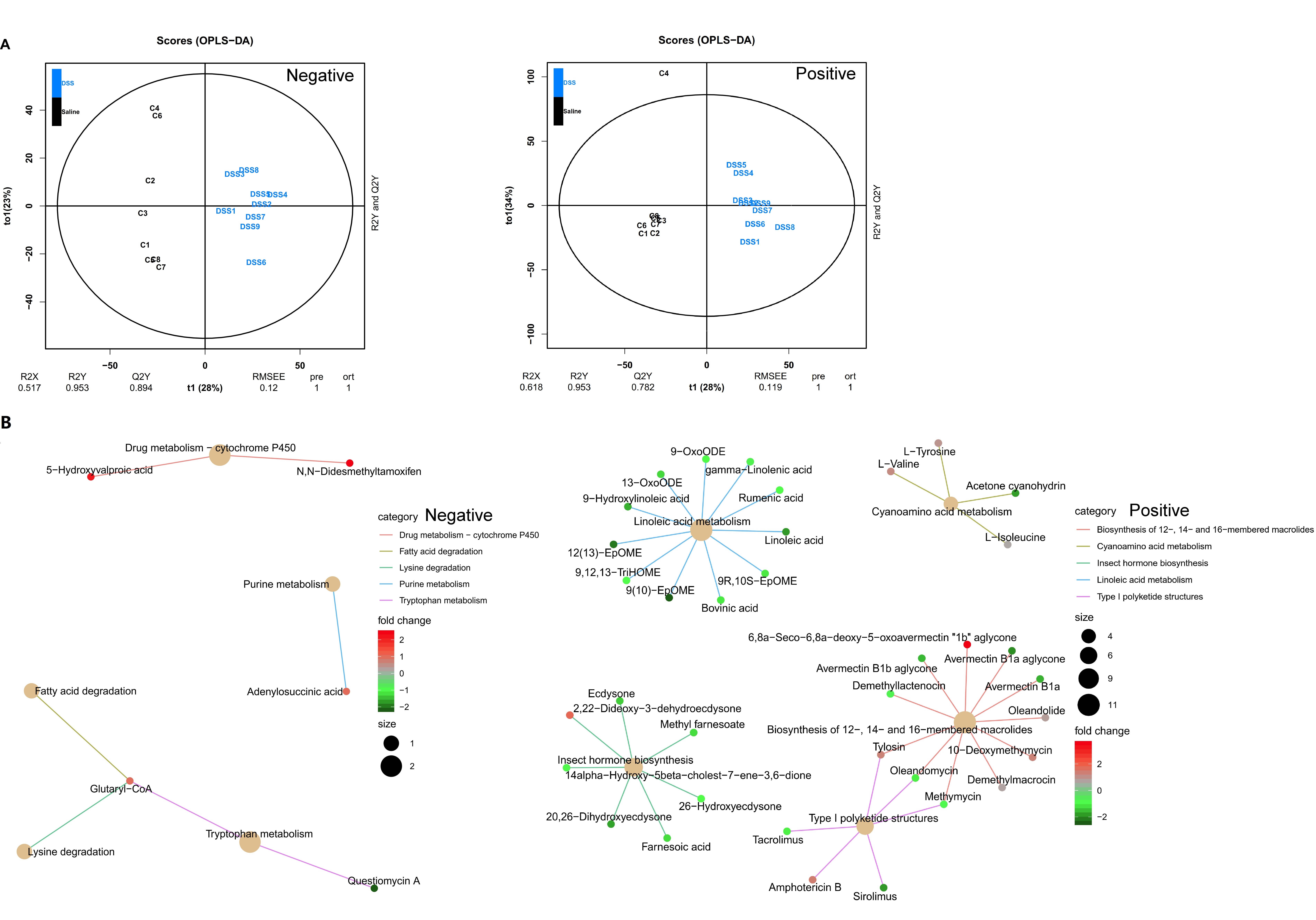

Supplement: Supplementary file 2 [file Image3.JPEG]

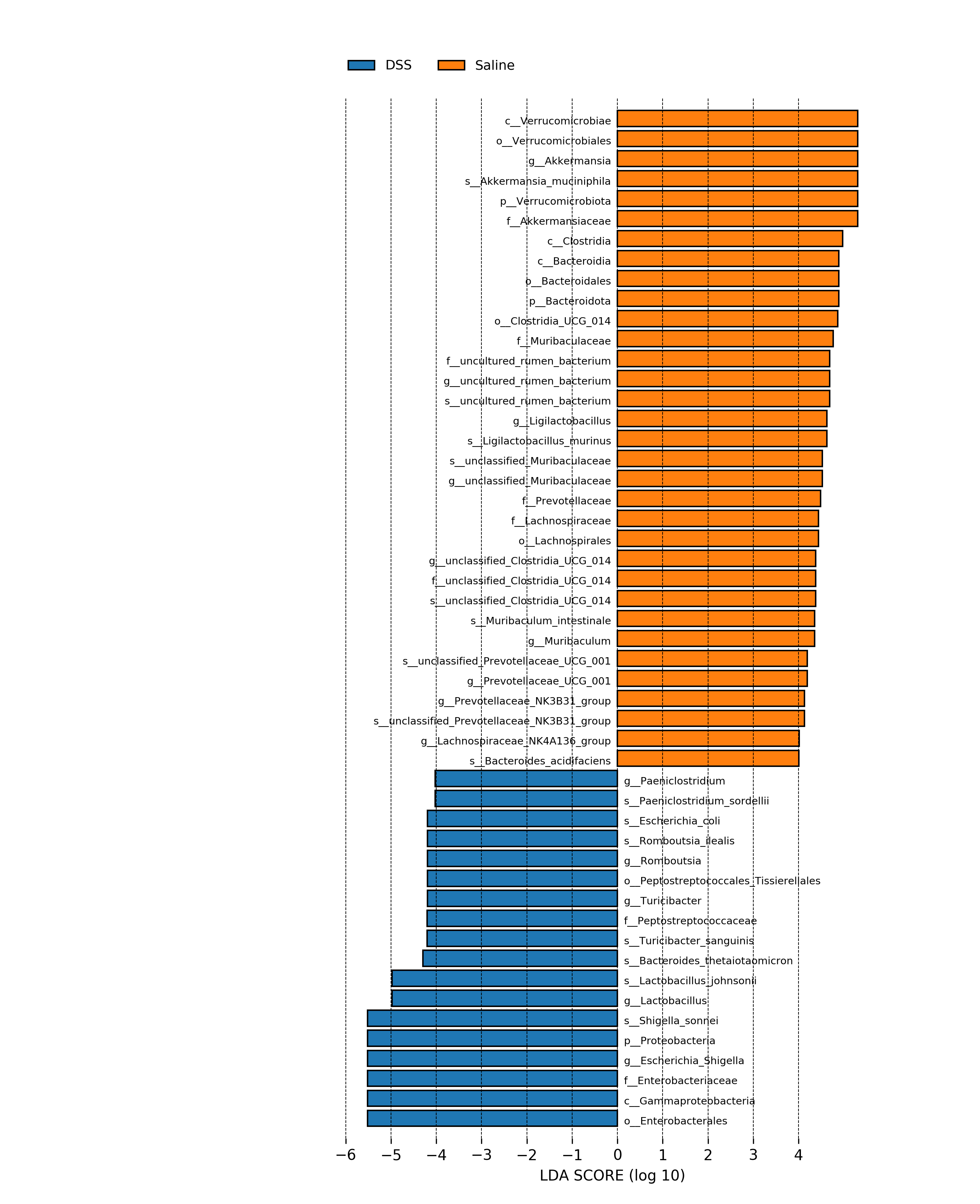

Supplement: Supplementary file 5 [file Image2.PNG]

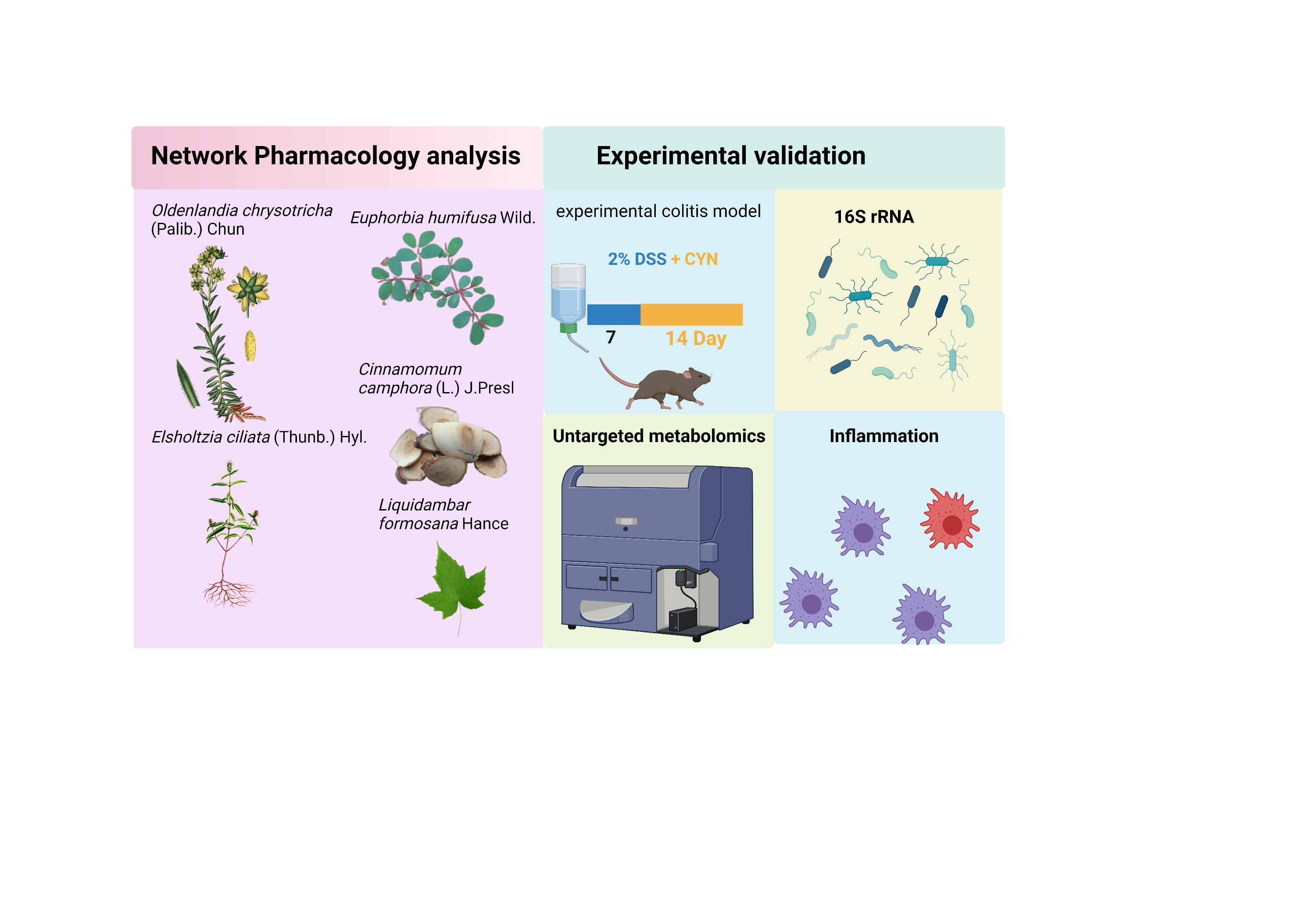

Supplement: Supplementary file 7 [file Image1.PNG]
